# Complete chloroplast genome sequence and phylogenetic analysis of *Spathiphyllum* 'Parrish'

**DOI:** 10.1371/journal.pone.0224038

**Published:** 2019-10-23

**Authors:** Xiao-Fei Liu, Gen-Fa Zhu, Dong-Mei Li, Xiao-Jing Wang

**Affiliations:** 1 Guangdong Provincial Key Laboratory of Biotechnology for Plant Development, College of Life Science, South China Normal University, Guangzhou, Guangdong, China; 2 Guangdong Key Lab of Ornamental Plant Germplasm Innovation and Utilization, Environmental Horticulture Research Institute, Guangdong Academy of Agricultural Sciences, Guangzhou, Guangdong, China; North Dakota State University, UNITED STATES

## Abstract

*Spathiphyllum* is a very important tropical plant used as a small, potted, ornamental plant in South China, with an annual output value of hundreds of millions of yuan. In this study, we sequenced and analyzed the complete nucleotide sequence of the *Spathiphyllum* 'Parrish' chloroplast genome. The whole chloroplast genome is 168,493 bp in length, and includes a pair of inverted repeat (IR) regions (IRa and IRb, each 31,600 bp), separated by a small single-copy (SSC, 15,799 bp) region and a large single-copy (LSC, 89,494 bp) region. Our annotation revealed that the *S*. 'Parrish' chloroplast genome contained 132 genes, including 87 protein coding genes, 37 transfer RNA genes, and 8 ribosomal RNA genes. In the repeat structure analysis, we detected 281 simple sequence repeats (SSRs) which included mononucleotides (223), dinucleotides (28), trinucleotides (12), tetranucleotides (11), pentanucleotides (6), and hexanucleotides (1), in the *S*. 'Parrish' chloroplast genome. In addition, we identified 50 long repeats, comprising 18 forward repeats, 13 reverse repeats, 17 palindromic repeats, and 2 complementary repeats. Single nucleotide polymorphism (SNP) and insertion/deletion (indel) analyses of the chloroplast genome of the *S*. 'Parrish' relative *S*. *cannifolium* revealed 962 SNPs in *S*. 'Parrish'. There were 158 indels (90 insertions and 68 deletions) in the *S*. 'Parrish' chloroplast genome relative to the *S*. *cannifolium* chloroplast genome. Phylogenetic analysis of five species found *S*. 'Parrish' to be more closely related to *S*. *kochii* than to *S*. *cannifolium*. This study identified the characteristics of the *S*. 'Parrish' chloroplast genome, which will facilitate species identification and phylogenetic analysis within the genus *Spathiphyllum*.

## Introduction

*Spathiphyllum* is a genus of approximately 41 species [1] of monocotyledonous flowering plants in the family Araceae, and is one of the most popular ornamental plants. Members of this genus are evergreen herbaceous perennial plants with large leaves that are 12–65 cm long and 3–25 cm wide. The flowers are produced in a spadix, surrounded by a white or green spathe that is 10–30 cm long. Because *Spathiphyllum* grows in different environments, interspecific hybridization occurs quite readily, which makes its genetic background complex. Moreover, interspecific hybridization makes it difficult to identify different varieties. Therefore, exploring a more effective way of differentiating closely related species of *Spathiphyllum* is necessary. Because chloroplast genomes are highly conserved, many studies have used chloroplast DNA markers to analyze phylogenetic relationships and population variation [2–4].

Chloroplasts possess a highly conserved [5,6] tetrad structure, containing two inverted repeat (IR) regions (IRa and IRb), a small single-copy (SSC) region and a large single-copy (LSC) region [6–8]. In addition to photosynthesis, chloroplast genome-encoded proteins are involved in other metabolic processes, such as responses to heat, drought, salt, and light [9]. By studying of chloroplast genomes, we can obtain a deeper understanding of plant biology, diversity, evolution and climatic adaptation, DNA barcoding and genetic engineering [9–15]. The rapid development of high throughput sequencing technologies has made the large-scale acquisition of chloroplast genomic sequences possible [16–18]. Over 800 complete chloroplast genome sequences, including 300 from crop and tree genomes, have been made available in the National Center for Biotechnology Information (NCBI) organelle genome database since 1986, when the first chloroplast genome sequence was reported [9,19]. To date, the chloroplast genome of only one species of *Spathiphyllum* (*Spathiphyllum kochii*) has been reported [20]. Unfortunately, no further analysis of molecular markers in the chloroplast genome of *S*. *kochii* has been published.

Slipped-strand mispairing, occurring in SSRs of 10 bp or longer, is the main mutational mechanism of SSR polymorphisms [18,21]. Chloroplast SSRs are highly efficient molecular markers and are often widely used in evolutionary studies, species identification, and population genetics [22–24]. However, there are few molecular marker studies of the genus *Spthiphyllum*. Only one article on molecular comparison of the genus *Spthiphyllum* was retrieved. Using amplified fragment length polymorphism (AFLP) markers with near-infrared fluorescence-labeled primers, this study analyzed genetic relatedness of 63 commercial cultivars and breeding lines [25]. Here, we report the whole chloroplast genome sequence of *S*. 'Parrish' and characterize its long repeats and simple sequence repeats (SSRs). The chloroplast genome of *S*. 'Parrish' and the chloroplast genomes of other members of Araceae are compared and analyzed. Furthermore, insertions and deletions, single nucleotide polymorphism (SNP), and phylogenetics are analyzed. Our report will provide useful information for further studies, help identify *Spathiphyllum* species, and provide insight into their evolutionary history.

## Materials and methods

### Plant material and DNA sequencing

*S*. 'Parrish' was planted at the Environmental Horticulture Research Institute, Guangdong Academy of Agricultural Sciences (N23°23', E113°23', Guangzhou, China). We first extracted the chloroplast genome DNA from young leaves of *S*. 'Parrish', and used ultrasonicator (Covaris M220, Covaris, Woburn, MA, USA) to divide the DNA into 300-500-bp fragments. Second, shotgun libraries were constructed according to the TruSeq^™^ DNA Sample Prep Kit for Illumina. Third, the Illumina HiSeq XTen (Biozeron, Shanghai, China) Sequencing Platform was used for PE150 sequencing. Some of the original data (raw data) produced with this method were of low quality. To improve the accuracy of the results of subsequent analyses, the original sequencing data were processed as follows: (1) the adapter sequence of reads was removed; (2) the bases containing non-AGCT nucleotides at the 5' end before shearing were removed; (3) the terminal end of reads with a low sequence quality was pruned (sequencing quality value less than Q20); (4) the reads containing 10% Ns were removed; and (5) adapters and small segments less than 50 bp in length after mass pruning were excluded.

At the same time, we used another method, single-molecule real-time (SMRT) circular consensus sequencing, to obtain the whole chloroplast genome of *S*. 'Parrish', following the standard protocol provided with the PacBio platform (Biozeron, Shanghai, China). To obtain more accurate assembly results, the original sequencing data were processed by filtering out the following: (1) polymerase reads whose length was less than 100 bp; (2) polymerase reads with a mass less than 0.80; (3) subreads extracted from polymerase reads and adapter sequences; and (4) subreads whose length was less than 500 bp.

### Chloroplast genome assembly and validation

First, SOAPdenovo (v2.04) [26] was used to preliminarily assemble the Illumina sequencing data. Second, the PacBio sequencing data were compared using BLASR (San Diego, CA, USA) [27]. To reduce the errors of single bases and insertions/deletions (indels) in the long PacBio sequences, the data were corrected according to the results of the comparison. The PacBio raw reads were pre-processed by trimming the adapter sequences, low quality (Q < 0.80) reads, short reads (length < 100 bp) and short subreads (length <500 bp). Finally, the PacBio clean data were used for the assembly.

NOVOPlasty (v2.7.2) software (https://github.com/ndierckx/NOVOPlasty) was used for chloroplast genome assembly. The *S*. *kochii* chloroplast genome was used as the reference genome for the assembly of *S*. 'Parrish' samples. The *rbcL* gene of the reference genome was used as a seed sequence. The other parameters were set to the defaults. Then, clean reads were compared with the scaffold obtained by assembly. The results were locally assembled and optimized by paired-end and overlap relations of reads. The gaps in the assembly results were repaired using GapCloser (v1.12, http://soap.genomics.org.cn/soapdenovo.html) software, with the default parameters. Finally, the reference genome was used to correct the location and direction of the four chloroplast partitions (LSC/IRa/SSC/IRb), and the initial position of the chloroplast assembly sequence was determined to obtain the final chloroplast genome sequence.

### Gene annotation and codon usage

The protein-coding, transfer RNA (tRNA) and ribosomal RNA (rRNA) genes of the chloroplast genome of *S*. 'Parrish' were predicted by DOGMA (http://dogma.ccbb.utexas.edu/) [28]software. The parameters were set as follows: (1) genetic code for Blastx: 11; (2) percent identity cutoff for protein-coding genes: 60; (3) percent identity cutoff for RNAs: 60; and (4) COVE threshold for mitochondrial tRNAs: 20. Then, the redundancy in the initial genes predicted by DOGMA was eliminated. The ends of the genes and the exon/intron boundaries were manually corrected to obtain a high-accuracy gene set, using the protein-coding genes of the reference genome as a reference. Using the *S*. *kochii* chloroplast genome as the reference genome, the genome of *S*. 'Parrish' was assembled. Finally, OrganellarGenomeDRAW software (http://ogdraw.mpimp-golm.mpg.de/cgi-bin/ogdraw.pl) [29] was used to display a circle map.

The degree of codon preference can be reflected by the relative probability of a particular codon in the synonymous codon encoding the corresponding amino acid. To obtain the codon preference value, relative synonymous codon usage (RSCU) was calculated by CUSP (EMBOSS v6.6.0.0) with default parameters.

### SSRs and long repeat structure

Microsatellite analysis of contig sequences was carried out with the MIcroSAtellite (MISA) identification tool [30]. The parameters (unit_size, min_repeats) were defined as follows: 1–10, 2–6, 3–5, 4–5, 5–5, and 6–5; the minimum distance between two SSRs was set to 100 bp. Parametric significance was met under the following conditions: 10 or more repeats of one base, 6 or more repeats of two bases, 5 or more repeats of three bases, 5 or more repeats of four bases, 5 or more repeats of five bases and 5 or more repeats of six bases. Additionally, when the distance between the two microsatellites was less than 100 bp, the two microsatellites formed a composite microsatellite. Finally, primers were designed for the SSR sequences by Primer3 (v.0.4.0, http://primer3.ut.ee).

Long repeats were detected by using REPuter (http://bibiserv.techfak.uni-bielefeld.de/reputer/). The minimum sequence length was 30 bp, and the editing distance was 3; searches were performed in four repetitive ways: (1) F: forward, (2) R: reverse, (3) C: complementary, and (4) P: palindromic.

### SNP and indel detection and annotation

Using MUMmer4 alignment software (Maryland, USA) [31], global alignment between each sample and reference sequence was carried out, the sites that differed between the sample sequence and the reference sequence were identified, and the potential SNP loci were detected through preliminary filtering. The 100-bp sequences on both sides of the reference sequence SNP loci were extracted, and the extracted sequence and assembly results were aligned using BLAT (v35, http://genome.ucsc.edu) software to verify the SNP loci [32]. If the length of the alignment was less than 101 bp, the unreliable SNP was removed; if the alignment was repeated many times, the SNP that was considered to be a duplicate was also removed, and finally, a reliable SNP was obtained.

The preliminary insertion/deletions (indels) results were obtained by comparing the samples with reference sequences using LASTZ (v1.03.54, http://www.bx.psu.edu/miller_lab/dist/README.lastz-1.02.00) software. Then the best comparison results were selected through axt_correction, axtSort and axtBest programs, and indel results were obtained preliminarily. Then, 150 bp upstream and downstream of the reference sequence indel locus were compared with the sequence reads of the sample by BWA (http://bio-bwa.sourceforge.net) software and SAMtools (http://samtools.sourceforge.net/), and reliable indels were obtained by filtering.

### Genome comparison

The whole chloroplast genomes of *S*. 'Parrish' (MK391158), *S*. *cannifolium* (MK372232) [33], *S*. *kochii* (KR270822) [20], *Dieffenbachia seguine* (KR262889), and *Pinellia ternata* (KR270823) were compared by mVISTA [34], with the annotation of *S*. 'Parrish' as the reference.

### Phylogenetic analysis

An evolutionary tree was constructed based on the population SNP matrix of the sample and reference genome. For each sample, all SNPs were linked in the same order to obtain FASTA format sequences of the same length, one of which was the reference sequence used as an input file for the construction of the evolutionary tree. An evolutionary tree was also constructed based on the core gene: a single-copy core gene identified by gene clustering was used to compare multiple protein sequences using MUSCLE (v3.8.31) software [35], and the results were used to construct an evolutionary tree. PhyML (v3.0, http://www.atgc-montpellier.fr/phyml/) and 1000 bootstraps were used to construct the phylogenetic tree with the maximum likelihood (ML) method [36]. Data files used in the phylogeny analysis has been added to the supplemental file (S1 and S2 Data).

The GenBank accession numbers for each plant species were as follows: *S*. 'Parrish' (MK391158), *S*. *cannifolium* (MK372232), *S*. *kochii* (KR270822), *D*. *seguine* (KR262889), *P*. *ternata* (KR270823), *Phoenix dactylifera* (GU811709), *Elaeis guineensis* (JF274081), *Lilium longiflorum* (KC968977), *Cocos nucifera* (KF285453), *Lemna minor* (NC_010109), *Typha latifolia* (NC_013823), *Colocasia esculenta* (NC_016753), *Fritillaria taipaiensis* (NC_023247), *Brassica napus* (NC_016734) and *Raphanus sativus* (NC_024469), with the last two species used as outgroups.

## Results and discussion

### Features of S. 'Parrish' chloroplast genome DNA

**Fig 1:**
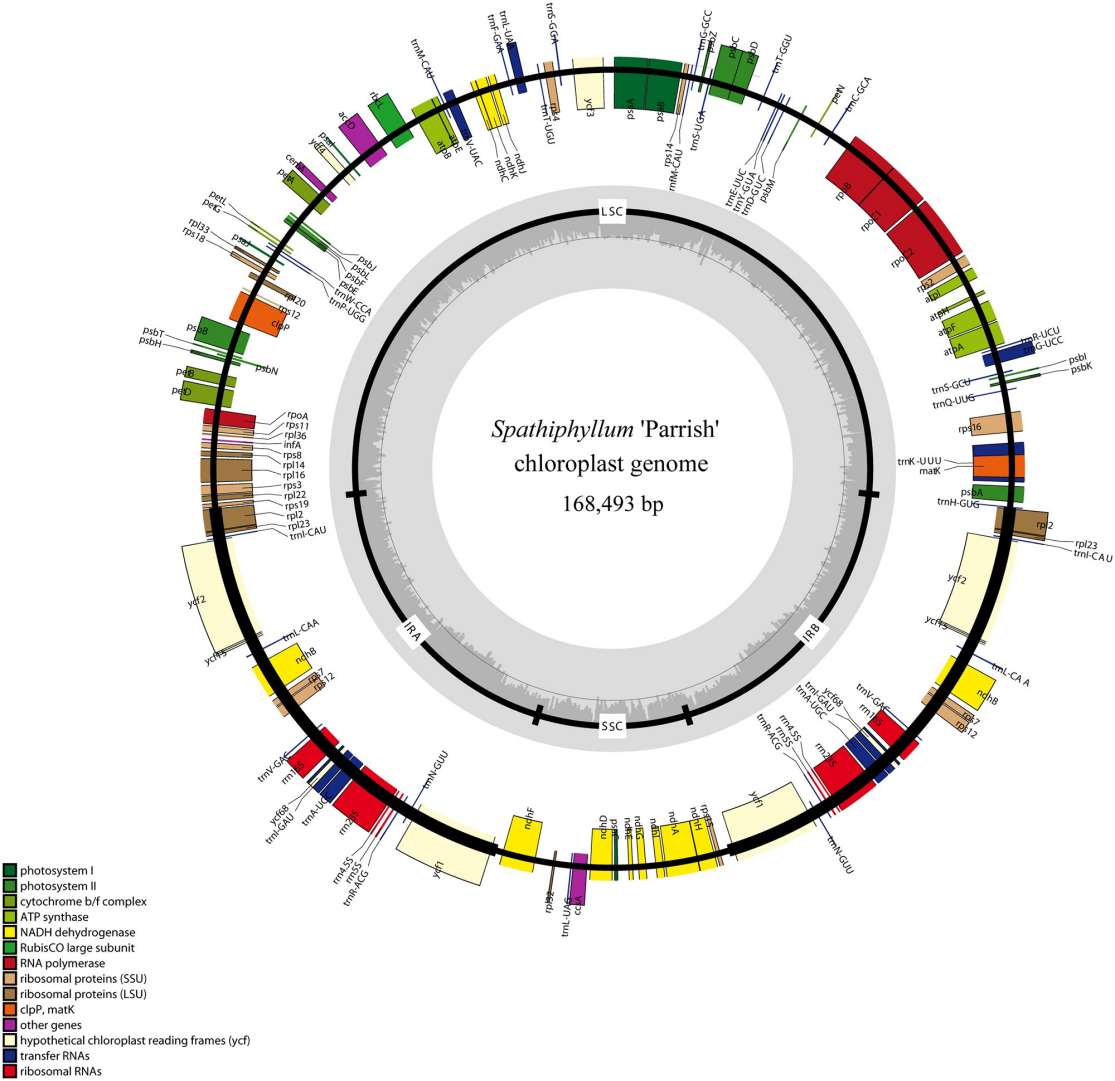
Gene map of *S*. 'Parrish'. Genes lying outside the circle are transcribed in a clockwise direction, whereas genes inside are transcribed in a counterclockwise direction. Different colors denote known functional groups. The GC and AT contents of the genome are denoted by dashed darker and lighter gray in the inner circle. LSC, SSC, and IR indicate large single-copy, small single-copy, and inverted repeat regions, respectively.

The length of the *S*. 'Parrish' chloroplast genome is 168,493 bp. The genome has a quadripartite structure with an SSC of 15,799 bp and an LSC of 89,494 bp, which are separated by two IR regions (IRa and IRb, each 31,600 bp) (Fig 1 and Table 1). The GC content of the overall chloroplast genome and the LSC, SSC, and IR regions is 36.19, 34.72, 29.35, and 39.98%, respectively (Table 1); these values are similar to those found for the genome of *S*. *kochii* [20]. The GC content of the two IR regions is higher than that of the LSC and SSC, which is a very common pattern in other plants [21], and this phenomenon is mostly attributable to rRNA genes and tRNA genes [37].

**Table 1:** Summary of the *S*. 'Parrish' chloroplast genome features.

Attribute	*S*. 'Parrish'
Genome size/GC content	168,493/36.19
Coding gene number/size	87/85,110
tRNA gene number/size	37/2,814
rRNA gene number/size	8/9,050
LSC size/percent/GC content	89,494/53.11/34.72
SSC size/percent/GC content	15,799/9.37/29.35
IR size/percent/GC content	31,600/18.75/39.98
Intron size/percent	17754/10.53
Intergentic spacer size/percent	53,685/31.86

The *S*. 'Parrish' chloroplast genome encodes 132 genes in total, comprising 87 protein-coding genes, 37 tRNA genes and 8 rRNA genes (Table 2). The IR region includes 7 protein-coding genes, 7 tRNA genes and 4 rRNA genes. The SSC contains 11 protein-coding genes and 1 tRNA gene, while the LSC contains 62 protein-coding genes and 22 tRNA genes (Fig 1).

**Table 2:** List of annotated genes in the *S*. 'Parrish' chloroplast genome.

Function	Genes
RNAs, transfer	*trnH-GUG*, *trnK-UUU* *, *trnQ-UUG*, *trnS-GCU*, *trnG-GCC* *, *trnR-UCU*, *trnC-GCA*, *trnD-GUC*, *trnY-GUA*, *trnE-UUC*, *trnT-UGU*, *trnS-UGA*, *trnG-UCC*, *trnfM-CAU*, *trnS-GCU*, *trnT-UGU*, *trnL-UAA* *, *trnF-GAA*, *trnV-UAC* *, *trnM-CAU*, *trnW-CCA*, *trnP-UGG*, *trnI-CAU*, *trnL-CAA*, *trnV-GAC*, *trnI-GAU* *, *trnA-UGC* *, *trnR-ACG*, *trnN-GUU*, *trnL-UAG*, *trnN-GUU*, *trnR-ACG*, *trnA-UGC* *, *trnI-GAU* *, *trnV-GAC*, *trnL-CAA*, *trnI-CAU*
RNAs, ribosomal	*rrn23* ^a^, *rrn16* ^a^, *rrn5* ^a^, *rrn4*.*5* ^a^,
Transcription and splicing	*rpoC1* *, *rpoC2*, *rpoA*, *rpoB*
Translation, ribosomal proteins	
Small subunit	*rps2*, *rps3*,*rps4*, *rps7*, *rps8*, *rps11*, *rps12* **, *rps14*, *rps15*, *rps16* *, *rps18*,*rps19*
Large subunit	*rpl2* *, *rpl14*, *rpl16* *, *rpl20*,*rpl22*,*rpl23*, *rpl32*, *rpl33*,*rpl36*
Photosynthesis	
ATP synthase	*atpE*, *atpB*, *atpA*, *atpF* *, *atpH*, *atpI*
Photosystem I	*psaI*, *psaJ*, *psaB*, *psaA*, *psaC*, *psaJ*, *ycf2*, *ycf3* **, *ycf4*
Photosystem II	*psbD*, *psbC*, *psbZ*, *psbT*, *psbH*, *psbK*, *psbI*, *psbJ*, *psbF*, *psbE*, *psbM*, *psbN*, *psbL*, *psbA*, *psbB*
Calvin cycle	*rbcL*
Cytochrome complex	*petN*, *petA*, *petL*, *petG*, *petB* *, *petD* *
NADH dehydrogenase	*ndhB* *, *ndhI*, *ndhK*, *ndhC*, *ndhF*, *ndhD*, *ndhG*, *ndhE*, *ndhA* *, *ndhH*, *ndhJ*
Others	*ycf1*, *ycf68*, *accD*, *cemA*, *ccsA*, *clpP* **, *matK*

* Genes containing one intron;

** Genes containing two introns;

^a^ Duplicated gene (genes present in the IR regions).

The frequency of codon usage was inferred based on the sequence of protein-coding genes and tRNA genes (Table 3). In total, 28,423 codons, which encoded all genes, were detected in *S*. 'Parrish'. Of these codons, 2,903 (10.21%) encode leucine, which is the most frequent amino acid in the chloroplast genome, and 333 (1.17%) encode cysteine, which is the least frequent.

**Table 3:** Codon usage in *S*. 'Parrish'.

Codon	Count	RSCU	Codon	Count	RSCU	Codon	Count	RSCU	Codon	Count	RSCU
UUU(F)	1054	1.29	UCU(S)	609	1.62	UAU(Y)	832	1.56	UGU(C)	256	1.54
UUC(F)	574	0.71	UCC(S)	389	1.03	UAC(Y)	234	0.44	UGC(C)	77	0.46
UUA(L)	882	1.82	UCA(S)	496	1.32	UAA(*)	36	1.24	UGA(*)	22	0.76
UUG(L)	602	1.24	UCG(S)	176	0.47	UAG(*)	29	1	UGG(W)	502	1
CUU(L)	614	1.27	CCU(P)	454	1.54	CAU(H)	523	1.51	CGU(R)	373	1.29
CUC(L)	207	0.43	CCC(P)	228	0.77	CAC(H)	172	0.49	CGC(R)	96	0.33
CUA(L)	411	0.85	CCA(P)	366	1.24	CAA(Q)	768	1.52	CGA(R)	377	1.31
CUG(L)	187	0.39	CCG(P)	133	0.45	CAG(Q)	241	0.48	CGG(R)	124	0.43
AUU(I)	1186	1.5	ACU(T)	572	1.55	AAU(N)	1075	1.53	AGU(S)	465	1.24
AUC(I)	444	0.56	ACC(T)	253	0.68	AAC(N)	334	0.47	AGC(S)	124	0.33
AUA(I)	747	0.94	ACA(T)	476	1.29	AAA(K)	1208	1.5	AGA(R)	590	2.05
AUG(M)	639	1	ACG(T)	177	0.48	AAG(K)	402	0.5	AGG(R)	171	0.59
GUU(V)	553	1.45	GCU(A)	643	1.78	GAU(D)	935	1.6	GGU(G)	611	1.31
GUC(V)	213	0.56	GCC(A)	223	0.62	GAC(D)	235	0.4	GGC(G)	174	0.37
GUA(V)	544	1.43	GCA(A)	421	1.17	GAA(E)	1129	1.49	GGA(G)	764	1.64
GUG(V)	216	0.57	GCG(A)	155	0.43	GAG(E)	388	0.51	GGG(G)	312	0.67

The chloroplast genome of *S*. 'Parrish' contains 19 intron-containing genes, including 6 tRNA genes and 13 protein-coding genes. *Ycf3* and *clpP* contain two introns, and the other 17 genes include one intron (Table 4). The intron (2,569 bp) of the *trnK-UUU* gene, which is the largest intron, includes the *matK* gene. The *rps12* gene is a trans-spliced gene with the 5’ end located in the LSC region and the duplicated 3’ ends in the IR regions. *Ycf3* is required for the stable accumulation of the photosystem I complex [38, 39]. The introns in the *S*. 'Parrish' chloroplast genome may be useful for further studies of the mechanism of photosynthesis evolution.

**Table 4:** The length of exons and introns in genes with introns in the *S*. 'Parrish' chloroplast genome.

Gene	Location	Exon I (bp)	Intron I (bp)	Exon II (bp)	Intron II (bp)	Exon III (bp)
*trnK-UUU*	LSC	42	2569	37		
*trnG-GCC*	LSC	24	746	48		
*trnL-UAA*	LSC	35	527	50		
*trnV-UAC*	LSC	37	597	38		
*trnI-GAU*	IR	42	943	35		
*trnA-UGC*	IR	38	805	35		
*rps12* *	LSC	26	542	232		126
*rps16*	LSC	197	1099	40		
*atpF*	LSC	386	829	154		
*rpoC1*	LSC	1639	725	455		
*ycf3*	LSC	141	787	209	777	124
*clpP*	LSC	275	649	277	701	66
*petB*	LSC	6	55	642		
*petD*	LSC	8	747	475		
*rpl16*	LSC	399	1195	9		
*rpl2*	IR	443	652	391		
*ndhB*	IR	778	650	782		
*ycf68*	IR	42	33	411		
*ndhA*	SSC	518	1136	562		

* The *rps12* gene is a trans-spliced gene with the 5’ end located in the LSC region and the duplicated 3’ ends in the IR regions.

Intron or gene gain or loss can be found in chloroplast genomes [8, 40–42] and may be significant during evolution. However, few studies have reported on the mechanism of photosynthesis evolution in *Spathiphyllum*. In this paper, we compared the chloroplast genome of *S*. 'Parrish' to that of other species of monocotyledons. These results provide a theoretical foundation for *Spathiphyllum* chloroplast genome research, breeding and molecular marker development.

### Long repeat and SSR analysis

**Fig 2:**
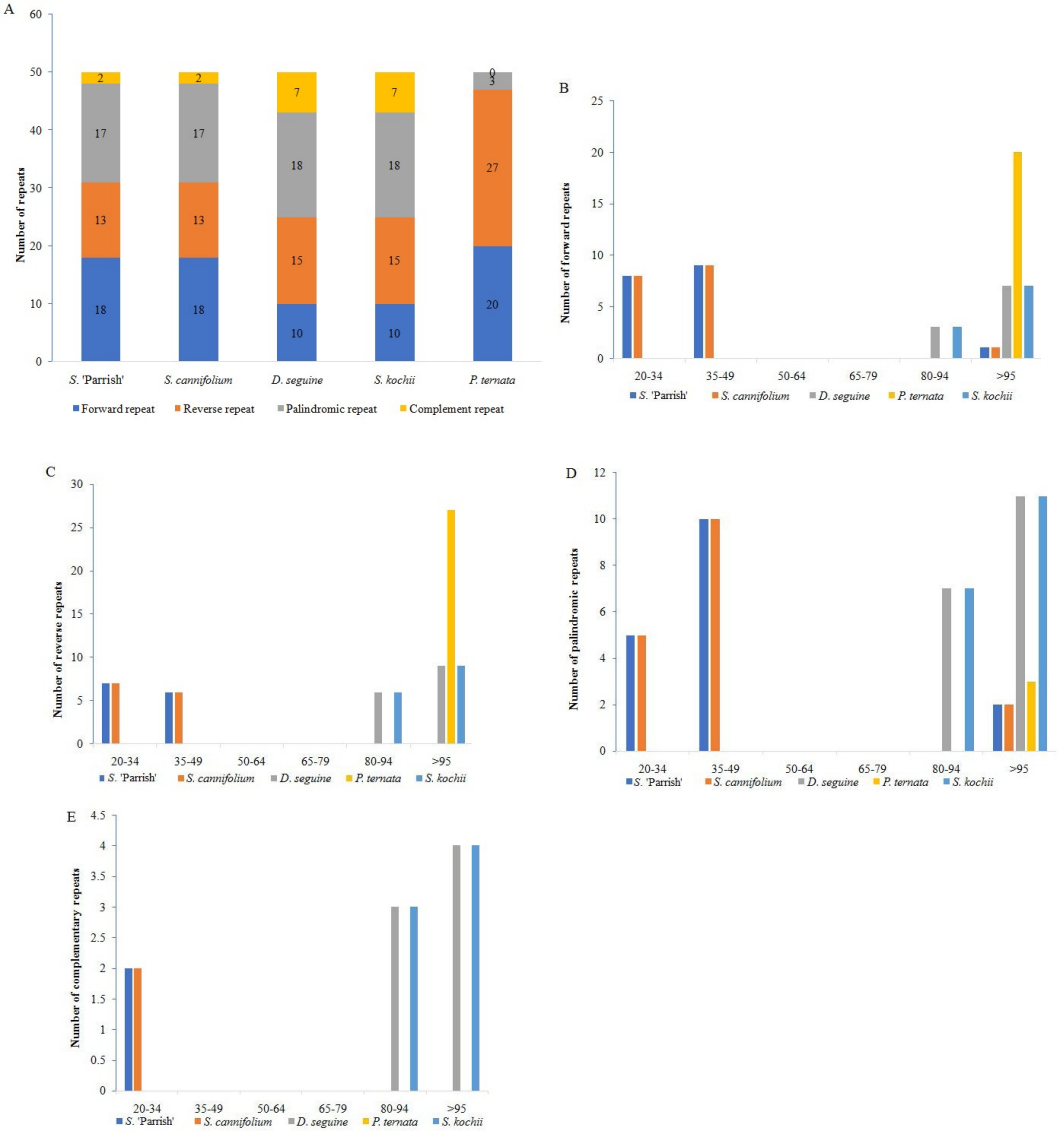
Analysis of repeated sequences in five Araceae chloroplast genomes. **(A)** Totals of four repeat types; **(B)** frequency of forward repeats by length; **(C)** frequency of reverse repeats by length; **(D)** frequency of palindromic repeats by length; **(E)** frequency of complementary repeats by length.

The *S*. 'Parrish' chloroplast genome includes 50 repeats in total, comprising 18 forward repeats, 13 reverse repeats, 17 palindromic repeats, and 2 complementary repeats (Fig 2 and S1 Table). Among these repeats, most of the forward repeats, reverse repeats, palindromic repeats, and complementary repeats are 20–49 bp in length (Fig 2B–2E). Similar repeat lengths were observed in *S*. *cannifolium* (Fig 2B–2E). In contrast, most of the repeats in *D*. *seguine*, *S*. *kochii*, and *P*. *ternata* are longer than 80 bp (Fig 2B–2E). However, the number of long repeats in *S*. *cannifolium*, *D*. *seguine*, *S*. *kochii*, and *P*. *ternata* is also 50 (Fig 2A and S2–S5 Tables).

**Fig 3:**
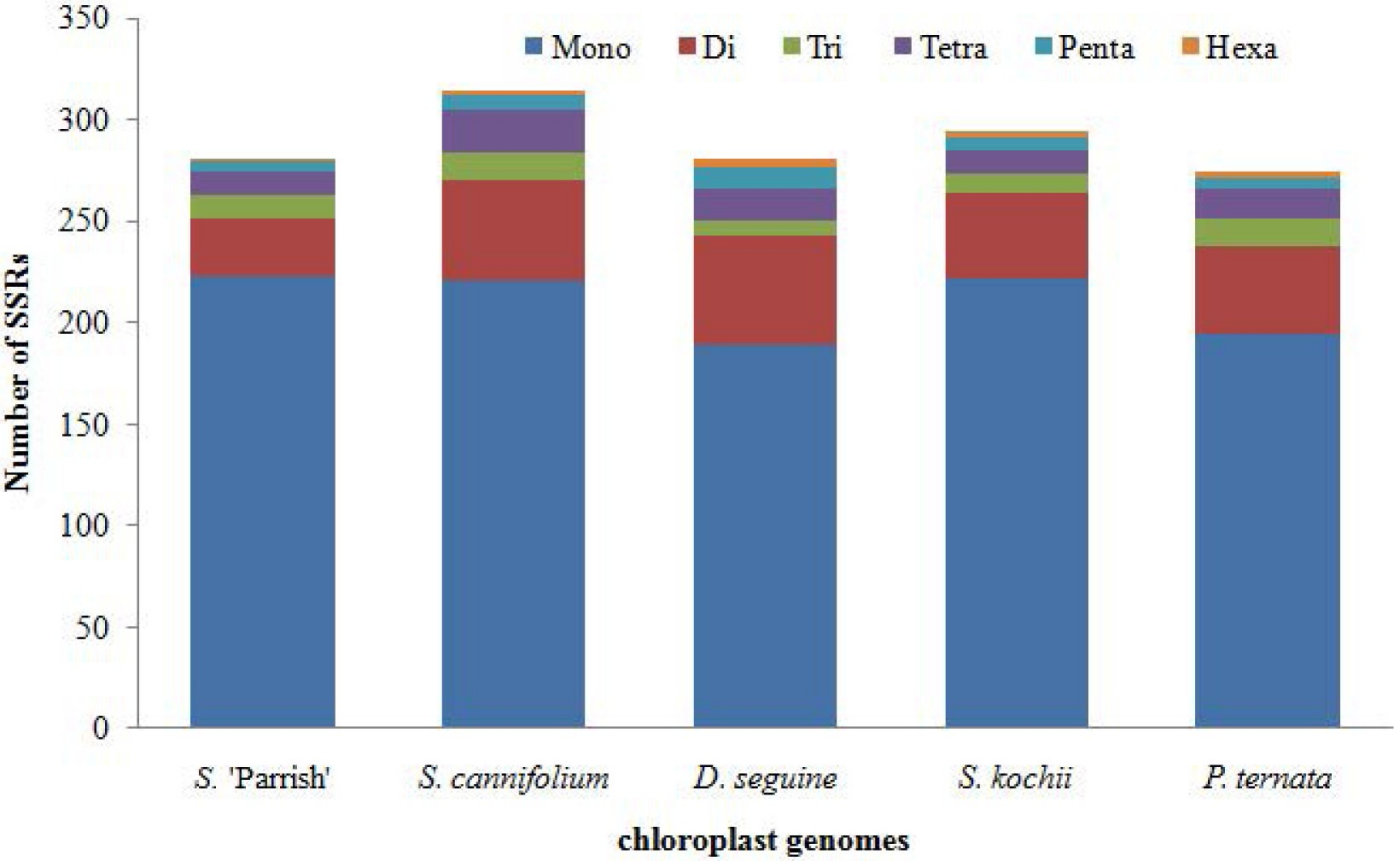
Analysis of SSRs in the five Araceae chloroplast genomes.

In this study, we detected 281 SSRs, which included 223 mononucleotides, 28 dinucleotides, 12 trinucleotides, 11 tetranucleotides, 6 pentanucleotides, and 1 hexanucleotide, in the chloroplast genome of *S*. 'Parrish' (Fig 3). Mononucleotides account for 97.8% of the SSRs in the *S*. 'Parrish' chloroplast genome. The number of SSRs in *S*. 'Parrish', *S*. *cannifolium*, *D*. *seguine*, *S*. *kochii*, and *P*. *ternata* is 281, 314, 281, 294 and 274, respectively (Fig 3). The hexanucleotide repeat content in *S*. ‘Parrish’ is the lowest among the five species (*S*. 'Parrish', 0.36%; *S*. *cannifolium*, 0.64%; *D*. *seguine*, 1.78%; *S*. *kochii*, 1.02%; and *P*. *ternata*, 1.09%). Mononucleotides are the most frequent repeat type in all of these species (*S*. 'Parrish', 79.36%; *S*. *cannifolium*, 70.38%; *D*. *seguine*, 67.26%; *S*. *kochii*, 75.51%; and *P*. *ternata*, 70.80%) (Fig 3). The findings of this study will help enable the use of chloroplast SSRs in the selection of germplasm for *Spathiphyllum* breeding.

### SNP and indel detection and annotation

Analysis of SNPs and indels in the chloroplast genome of *S*. 'Parrish' relative to that of *S*. *cannifolium* revealed 962 SNPs in *S*. 'Parrish'. Of these SNPs (S6 Table), 704 were located in intergenic regions, representing the most frequently occurring mutations, and the coding regions included 134 synonymous SNPs, 123 nonsynonymous SNPs, and 1 stop mutation. There were 158 indels, including 90 insertions and 68 deletions, in the *S*. 'Parrish' chloroplast genome relative to the *S*. *cannifolium* chloroplast genome (S1 Fig and S7 Table). Of these 158 indels, 57 (36.08%) were single-base indels, which differed from the numbers in maize and sugarcane [8, 43, 44]. It indicated that the nucleotide substitution events in the chloroplast genomes of *Spathiphyllum* species were more than that between species of *Oryza* and *Kaempferia*. Comparative analysis of chloroplast genomes found 159 SNPs between *Oryza nivara* and *O*. *sativa* [45], 536 SNPs and 107 indels between *Kaempferia Galanga* and *Kaempferia Elegans* [46]. The analysis of these SNPs and indels molecular markers can provide theoretical basis for species identification in the future.

### IR contraction and expansion in the S. 'Parrish' chloroplast genome

**Fig 4:**
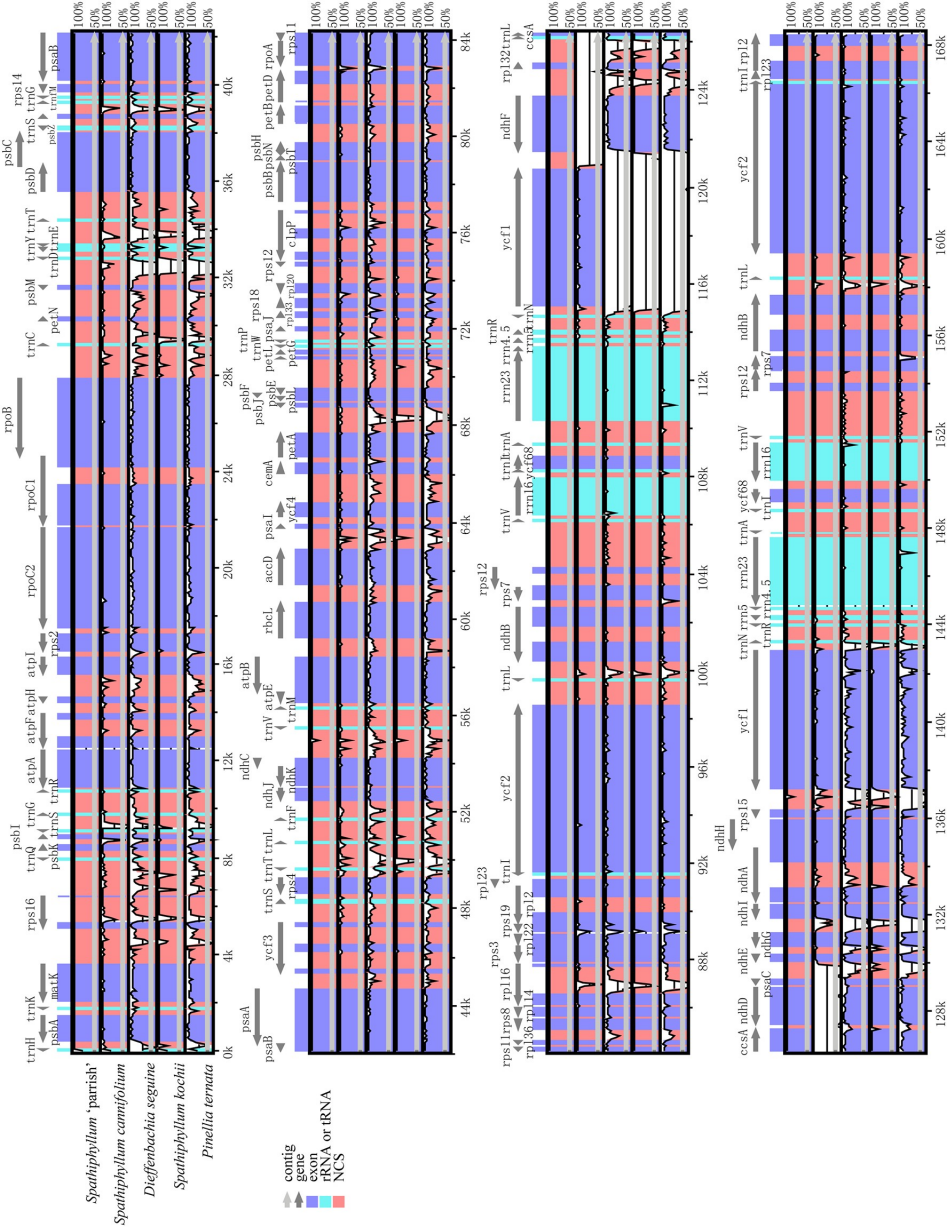
Comparison of the LSC, SSC, and IR regions among five chloroplast genomes. Boxes above the main line indicate the adjacent border genes. The figure is not to scale with respect to sequence length, and shows only relative changes at or near the IR/SC borders.

Contraction and expansion at the borders of IR regions are common evolutionary events and are the main explanations for the size variation among chloroplast genomes [49, 50]. Detailed comparisons of the four junctions IRa-LSC, IRb-SSC, IRa-SSC, and IRb-LSC among five Araceae chloroplast genomes (*S*. 'Parrish', *S*. *cannifolium*, *D*. *seguine*, *S*. *kochii*, and *P*. *ternata*) are presented in Fig 3. The *rps19* gene is located in the LSC region 30, 30, 47, 39, and 41 bp away from the LSC-IRa border in these five Araceae chloroplast genomes, respectively. The *rpl2* gene is located in the IR regions, and the IRa region of the five species contains 51, 51, 40, 42, and 47 bp, while the IRb region contains 52, 52, 46, 43, and 48 bp, respectively. The *trnH-GUG* gene is located in the LSC region 0, 77, 431, 63, and o bp away from the IRb-LSC border in these species, respectively (Fig 4). The length of the IR regions may be the main reason for the differences among the five Araceae chloroplast genomes (*S*. 'Parrish', 31,603 bp; *S*. *cannifolium*, 31,457 bp; *D*. *seguine*, 25,256 bp; *S*. *kochii*, 25,281 bp; and *P*. *ternata*, 25,625 bp).

### Comparative chloroplast genome analysis

**Fig 5:**
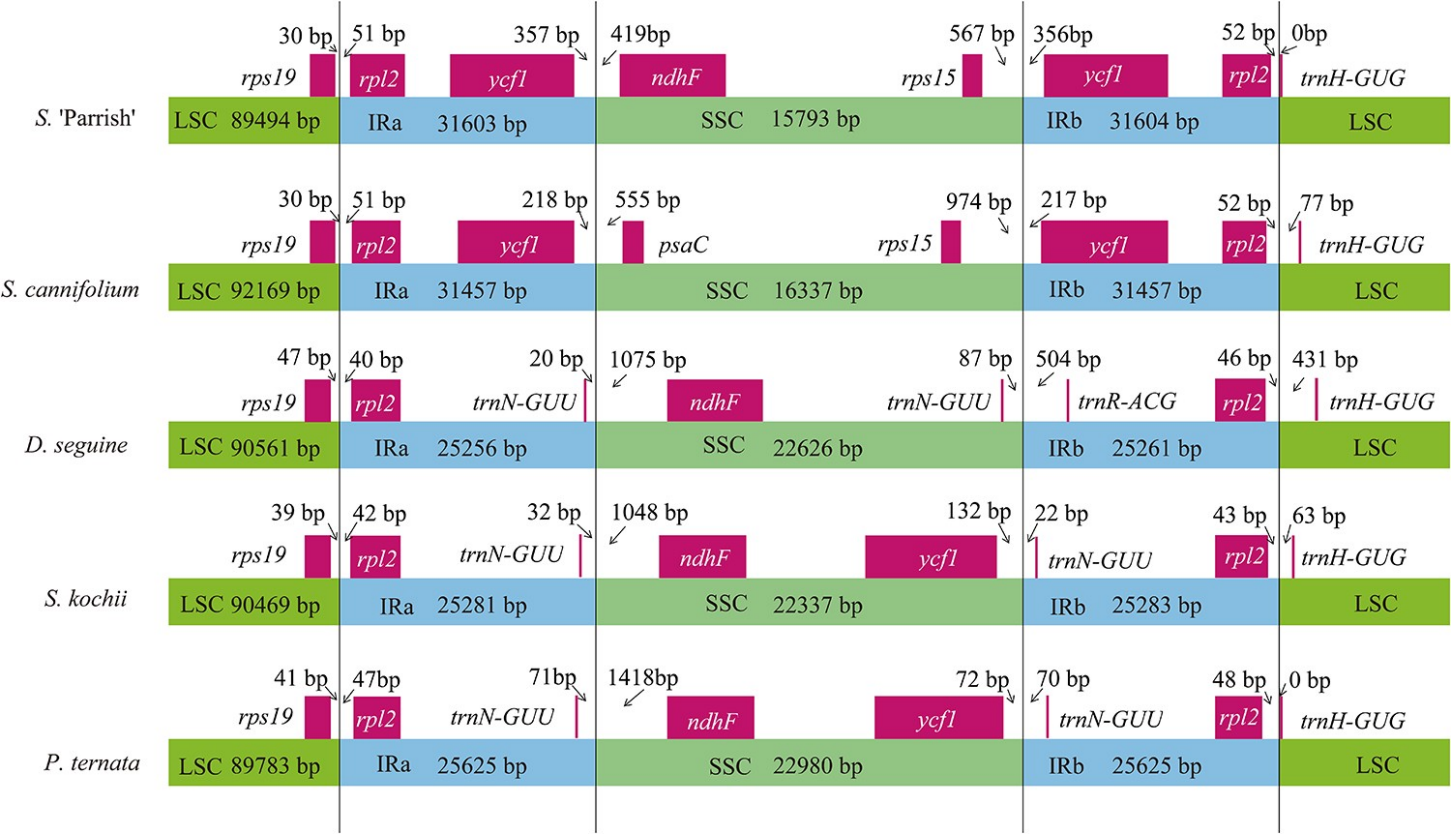
Comparison of five chloroplast genomes using mVISTA. Gray arrows and thick black lines above the alignment indicate gene orientation. Purple bars represent exons, blue bars represent rRNA or tRNA genes, and pink bars represent noncoding sequences (NCS). The y-axis represents the percent identity (shown: 50–100%).

Comparative analysis of chloroplast genomes is an essential step in genomics [47, 48]. A comparison of the structural differences among Araceae chloroplast genomes indicates that the chloroplast genome of *S*. *kochii* is the smallest (Fig 4; *S*. 'Parrish', 168,493 bp; *S*. *cannifolium*, 171,420 bp; *D*. *seguine*, 163,704 bp; *S*. *kochii*, 163,368 bp; and *P*. *ternata*, 164,013 bp). To explain the level of genome divergence, the whole sequence identity of the five Araceae chloroplast genomes was calculated using mVISTA with *S*. 'Parrish' as a reference (Fig 5). The IR (A/B) regions exhibited less divergence than the SSC and LSC regions. In addition, the noncoding regions showed more differences than the coding regions. Except for the noncoding regions, the most highly divergent regions between *S*. 'Parrish' and *S*. *cannifolium* were mainly in *ndhF*-*ndhE* in the IRa and SSC regions (Fig 5), the length of which was approximately 10 kb. Except for the noncoding regions, the most frequently divergent regions between *S*. 'Parrish' and *S*. *kochii* were mainly in the coding regions of the *ycf1* sequence in the IRa and SSC regions (Fig 5), the length of which was approximately 7 kb. The difference in regional structure between the two segments may be responsible for the closer relationship between *S*. 'Parrish' and *S*. *kochii* than between *S*. 'Parrish' and *S*. *cannifolium*.

### Phylogenetic analysis

**Fig 6:**
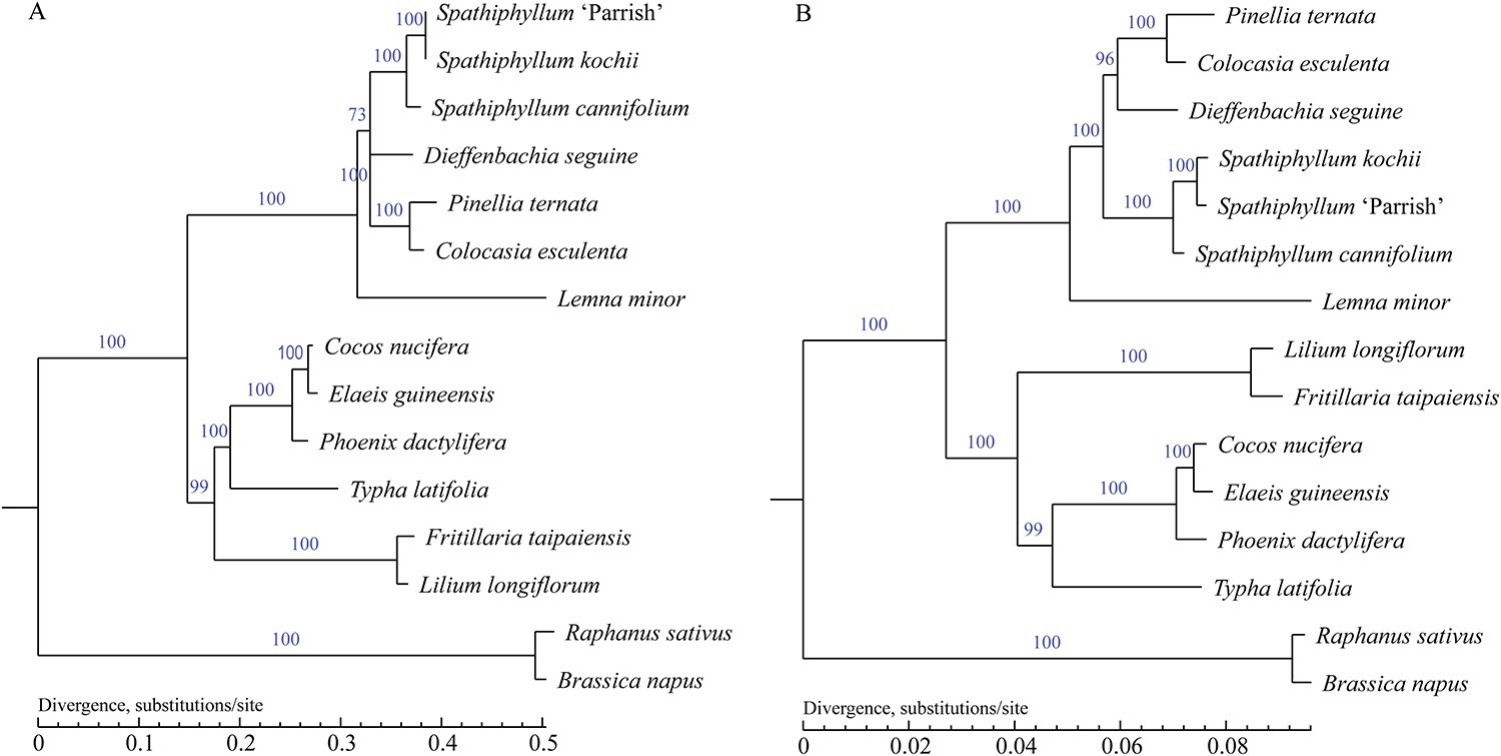
**(A) Phylogenetic tree reconstruction of 15 species based on sequences from whole chloroplast genomes by the maximum likelihood method. (B) Phylogenetic tree derived from all protein-coding genes from 15 species by the maximum likelihood method**. *Raphanus sativus* and *Brassica napus* were used as outgroups. All bootstrap supports are indicated near the node.

The complete chloroplast genome of *S*. 'Parrish' provides information that can be used to analyze the phylogenetic relationships of *S*. 'Parrish' with 15 other monocots. Multiple sequence alignment was performed using the whole chloroplast genome (Fig 6A) and the protein-coding genes (Fig 6B) in 15 monocots. The *B*. *napus* and *R*. *sativus* chloroplast genomes were used as outgroups. We used ML to construct a phylogenetic tree. In the tree, *S*. 'Parrish' was closer to *S*. *kochii* than to *S*. *cannifolium*. These results (Fig 6A and 6B) suggest that the two methods produce similar multiple sequence alignments, and the phylogenetic tree analysis shows that the chloroplast genome sequence is useful for species identification and genetics.

The difference in scale causes a difference in the alignment of the protein coding sequence and whole chloroplast genome. Second, we performed an alignment analysis on the complete sequences of three samples of *S*. 'Parrish' (MK391158), *S*. *cannifolium* (MK372232), *S*. *kochii* (KR270822), and found that the sequence similarity of the three chloroplast genomes was 99.53% (S2 Fig). The percentage system was shown on evolutionary branches and the difference in scale causes a difference in the alignment of the protein coding sequence and whole chloroplast genome.

## Conclusions

In this study, we reported and analyzed the complete chloroplast genome of *S*. 'Parrish', which is one of the most popular ornamental plants worldwide. A comparison of the structure of the Araceae chloroplast genomes revealed that the IRa and SSC regions were more divergent than the other two regions, and the noncoding regions showed more differences than the coding regions. In the repeat structure analysis, we detected 281 SSRs, which included 223 mononucleotides, 28 dinucleotides, 12 trinucleotides, 11 tetranucleotides, 6 pentanucleotides, and 1 hexanucleotide, in the *S*. 'Parrish' chloroplast genome. In addition, 50 long repeats, comprising 18 forward repeats, 13 reverse repeats, 17 palindromic repeats, and 2 complementary repeats, were identified. Analysis of SNPs and indels in the *S*. 'Parrish' chloroplast genome relative to the *S*. *cannifolium* chloroplast genome revealed 962 SNPs and 158 indels in the *S*. 'Parrish' chloroplast genome. Phylogenetic analysis among five species found *S*. 'Parrish' to be more closely related to *S*. *kochii* than to *S*. *cannifolium*. The results of this study provide an assembly of the whole chloroplast genome of *S*. 'Parrish' and information on its divergence from the chloroplast genome of other members of *Spathiphyllum*, which might be useful for future breeding and biological discoveries.

## Supporting information

S1 Fig Size and number of indels in *S*. 'Parrish' relative to that of *S*. *cannifolium* genome. (TIF)

S2 Fig Alignment results of the full sequence of three samples of *Spathiphyllum*. (TIF)

S1 Table Long repeat in the chloroplast genome of *S*. 'Parrish'. (XLS)

S2 Table Long repeat in the chloroplast genome of *S*. *cannifolium*. (XLSX)

S3 Table Long repeat in the chloroplast genome of *D*. *seguine*. (XLS)

S4 Table Long repeat in the chloroplast genome of *S*. *kochii*. (XLS)

S5 Table Long repeat in the chloroplast genome of *P*. *ternata*. (XLS)

S6 Table SNPs in *S*. 'Parrish' relative to that of *S*. *cannifolium* genome. (XLS)

S7 Table Indels in *S*. 'Parrish' relative to that of *S*. *cannifolium* genome. (XLS)

S1 Data Data for whole chloroplast genome sequence comparison. (FA)

S2 Data Data for protein coding sequence comparison. (FA)

## References

[1] Mayo SJ, Bogner J, Boyce PC. The genera of Araceae[M]. Royal Botanic Gardens Kew, UK 1997; 110.

[2] Manos PS, Cannon CH, Oh SH. Phylogenetic relationships and taxonomic status of the paleoendemic Fagaceae of western North America: Recognition of a new genus, *Notholithocarpus*. Madroño. 2008; 55, 181–190.

[3] Oh SH, Manos PS. Molecular phylogenetics and cupule evolution in Fagaceae as inferred from nuclear crabs claw sequences. Taxon. 2008; 57, 434–451.

[4] Li X, Li Y, Zang M, Li M, Fang Y. Complete Chloroplast Genome Sequence and Phylogenetic Analysis of *Quercus acutissim*a. Int. J. Mol. Sci. 2018; 19, 2443. 10.3390/ijms19082443 PMC6121628 30126202

[5] Ravi V, Khurana JP, Tyagi AK, Khurana P. An update on chloroplast genomes. Plant Syst Evol. 2008; 271:101–122.

[6] Jansen RK, Raubeson LA, Boore JL, dePamphilis CW, Chumley TW, Haberle RC, et al Methods for obtaining and analyzing chloroplast genome sequences. Methods Enzymol. 2005; 395: 348–384. 10.1016/S0076-6879(05)95020-9 15865976

[7] Wu C, Lai Y, Lin C, Wang Y, Chaw S. Evolution of reduced and compact chloroplastgenomes (chloroplastDNAs) in gnetophytes: selection toward a lower-cost strategy. Mol Phylogenet Evol. 2009; 52(1):115–124 10.1016/j.ympev.2008.12.026 19166950

[8] Kong W, Yang J. The complete chloroplast genome sequence of Morus mongolica and a comparative analysis within the Fabidae clade. Curr Genet. 2016; 62, 165–172. 10.1007/s00294-015-0507-9 26205390

[9] Daniell H, Lin CS, Chang WJ. Chloroplast genomes: diversity, evolution, and applications in genetic engineering. Genome Biol. 2016; 17, 134 10.1186/s13059-016-1004-2 27339192 PMC4918201

[10] Cazzonelli CI. Carotenoids in nature: Insights from plants and beyond. Funct. Plant Biol. 2011, 38, 833–847. 10.1071/FP11192 32480941

[11] Bobik K, Burch-Smith TM. Chloroplast signaling within, between and beyond cells. Front. Plant Sci. 2015, 6, 781 10.3389/fpls.2015.00781 26500659 PMC4593955

[12] Baczkiewicz A, Szczecinska M, Sawicki J, Stebel A, Buczkowska K. DNA barcoding, ecology and geography of the cryptic species of Aneura pinguis and their relationships with Aneura maxima and Aneura mirabilis (Metzgeriales, Marchantiophyta). PLoS ONE 2017, 12, e0188837 10.1371/journal.pone.0188837 29206876 PMC5716573

[13] Kang Y, Deng Z, Zang R, Long W. DNA barcoding analysis and phylogenetic relationships of tree species in tropical cloud forests. Sci. Rep. 2017, 7, 12564 10.1038/s41598-017-13057-0 28970548 PMC5624878

[14] Song Y, Chen Y, Lv J, Xu J, Zhu S, Li M, Chen N. Development of Chloroplast Genomic Resources for Oryza Species Discrimination. Front. Plant Sci. 2017, 8, 1854 10.3389/fpls.2017.01854 29118779 PMC5661024

[15] Liu X, Zhou B, Yang H, Li Y, Yang Q, Lu Y, Gao Y. Sequencing and Analysis of *Chrysanthemum carinatum* Schousb and *Kalimeris indica*. The Complete Chloroplast Genomes Reveal Two Inversions and *rbcL* as Barcoding of the Vegetable. Molecules. 2018, 23, 1358. 10.3390/molecules23061358 PMC6099409 29874832

[16] Hui L, Xie H, Jiang Z, Li C, Zhang G. Photosynthetic response of potted Quercus acutissima Carruth seedlings under different soil moisture conditions. Sci. Soil Water Conserv. 2013; 11, 93–97.

[17] Shen X, Wu M, Liao B, Liu Z, Bai R, Xiao S, et al Complete chloroplast genome sequence and phylogenetic analysis of the medicinal plant Artemisia annua. Molecules. 2017; 22, 1330. 10.3390/molecules22081330 PMC6152406 28800082

[18] Guo S, Guo L, Zhao W, Xu J, Li Y, Zhang X, et al Complete chloroplast genome sequence and phylogenetic analysis of *Paeonia ostii*. Molecules. 2018; 23, 246. 10.3390/molecules23020246 PMC6017096 29373520

[19] Shinozaki K, Ohme M, Tanaka M, Wakasugi T, Hayashida N, Matsubayashi T, et al The complete nucleotide sequence of the tobacco chloroplast genome: its gene organization and expression. EMBO J. 1986; 5:2043–9. 16453699 10.1002/j.1460-2075.1986.tb04464.x PMC1167080

[20] Han L, Wang B, Wang ZZ. The complete chloroplast genome sequence of *Spathiphyllum kochii*. Mitochondrial DNA A DNA Mapp Seq Anal. 2016, 27(4):2973–4. 10.3109/19401736.2015.1060466 26134343

[21] Asaf S, Khan AL, Khan MA, Waqas M, Kang SM, Yun BW, et al Chloroplast genomes of Arabidopsis halleri ssp. gemmifera and Arabidopsis lyrata ssp. petraea: Structures and comparative analysis. Sci. Rep. 2017; 7, 7556 10.1038/s41598-017-07891-5 28790364 PMC5548756

[22] Mohammad-Panah N, Shabanian N, Khadivi A, Rahmani M.-S, Emami A. Genetic structure of gall oak (Quercus infectoria) characterized by nuclear and chloroplast SSR markers. Tree Genet. Genomes. 2017; 13, 70–82.

[23] Park SH, Sang IP, Gil J, Hwangbo K, Um Y, Kim HB, et al Development of Chloroplast SSR Markers to Distinguish Codonopsis Species. Korean Soc. Hortic. Sci. 2017; 5, 207–208.

[24] Zeng J, Chen X, Wu XF, Jiao FC, Xiao BG, Li YP, et al Genetic diversity analysis of genus Nicotiana based on SSR markers in chloroplast genome and mitochondria genome. Acta Tab. Sin. 2016; 22, 89–97.

[25] Chen J, Henny RJ, Devanand PS, Chao CCT. Genetic Relationships of Spathiphyllum Cultivars Analyzed by AFLP Markers. HortScience: a publication of the American Society for Horticultural Science 41(4) 7 2006 with152.

[26] Luo R, Liu B, Xie Y, Li Z, Huang W, Yuan J, et al SOAPdenovo2: An empirically improved memory-efficient short-read de novo assembler. Gigascience. 2012, 1, 18–24. 10.1186/2047-217X-1-18 23587118 PMC3626529

[27] Chaisson MJ, Tesler G. Mapping single molecule sequencing reads using basic local alignment with successive refinement (BLASR): Application and theory. BMC Bioinform. 2012, 13, 238. 10.1186/1471-2105-13-238 PMC3572422 22988817

[28] Wyman SK, Jansen RK, Boore JL. Automatic annotation of organellar genomes with DOGMA. Bioinformatics, 2004, 20, 3252–3255. 10.1093/bioinformatics/bth352 15180927

[29] Lohse M, Drechsel O, Bock R. Organellar Genome DRAW (OGDRAW): A tool for the easy generation of high-quality custom graphical maps of plastid and mitochondrial genomes. Curr. Genet. 2007, 52, 267–274. 10.1007/s00294-007-0161-y 17957369

[30] MISA-MIcroSAtellite identification tool. http://pgrc.ipk-gatersleben.de/misa/ (accessed on 20 September 2017)

[31] Marcais G, Delcher AL, Phillippy AM, Coston R, Salzberg SL, et al MUMmer4: A fast and versatile genome alignment system. PLoS Comput. Biol. 2018,14, e1005944 10.1371/journal.pcbi.1005944 29373581 PMC5802927

[32] Bhagwat M, Young L, Robison RR. Using BLAT to find sequence similarity in closely related genomes. Curr. Protoc. Bioinform. 2012, 010, Unit10.8. 10.1002/0471250953.bi1008s37 PMC4101998 22389010

[33] Liu XF, Zhu GF, Li DM, Wang XJ. The complete chloroplast genome sequence of Spathiphyllumcannifolium, Mitochondrial DNA Part B, 2019, 4:1,

[34] Frazer KA, Pachter L, Poliakov A, Rubin EM, Dubchak I. VISTA: Computational tools for comparative genomics. Nucleic Acids Res. 2004, 32, 273–279. 10.1093/nar/gkh458 PMC441596 15215394

[35] Edgar RC. MUSCLE: multiple sequence alignment with high accuracy and high throughput. Nucleic acids research, 2004, 32(5): 1792–1797. 10.1093/nar/gkh340 15034147 PMC390337

[36] Guindon S, Dufayard JF, Lefort V. New algorithms and methods to estimate maximum-likelihood phylogenies: assessing the performance of PhyML 3.0. Systematic biology, 2010, 59(3): 307–321. 10.1093/sysbio/syq010 20525638

[37] He Y, Xiao H, Deng C, Xiong L, Yang J, Peng C. The complete chloroplast genome sequences of the medicinal plant Pogostemon cablin. Int. J. Mol. Sci. 2016; 17, 820. 10.3390/ijms17060820 PMC4926354 27275817

[38] Boudreau E, Takahashi Y, Lemieux C, Turmel M, Rochaix JD. The chloroplast ycf3 and ycf4 open reading frames of Chlamydomonas reinhardtii are required for the accumulation of the photosystem I complex. EMBO J. 1997; 16, 6095–6104. 10.1093/emboj/16.20.6095 9321389 PMC1326293

[39] Naver H, Boudreau E, Rochaix JD. Functional studies of *Ycf3*: Its role in assembly of photosystem I and interactions with some of its subunits. Plant Cell. 2001; 13, 2731–2745. 10.1105/tpc.010253 11752384 PMC139485

[40] Guisinger MM, Chumley TW, Kuehl JV, Boore JL, Jansen RK. Implications of the plastid genome sequence of Typha (Typhaceae, Poales) for understanding genome evolution in Poaceae. J. Mol. Evol. 2010; 70, 149–166. 10.1007/s00239-009-9317-3 20091301 PMC2825539

[41] Jansen RK, Cai Z, Raubeson LA, Daniell H, Depamphilis CW, Leebensmack J, et al Analysis of 81 genes from 64 plastid genomes resolves relationships in angiosperms and identifies genome-scale evolutionary patterns. Proc. Natl. Acad. Sci. USA. 2007; 104, 19369–19374. 10.1073/pnas.0709121104 18048330 PMC2148296

[42] Ueda M, Fujimoto M, Arimura S, Murata J, Tsutsumi N, Kadowaki K. Loss of the rpl32 gene from the chloroplast genome and subsequent acquisition of a preexisting transit peptide within the nuclear gene in Populus. Gene. 2007; 402, 51–56. 10.1016/j.gene.2007.07.019 17728076

[43] Yamane K, Yano K, Kawahara T. Pattern and rate of indels evolution inferred from whole chloroplast intergenic regions in sugarcane, maize and rice. DNA Res. 2006; 13(5):197–204. 10.1093/dnares/dsl012 17110395

[44] Carbonell-Caballero J, Alonso R, Ibanez V, Terol J, Talon M, Dopazo J. A phylogenetic analysis of 34 chloroplast genomes elucidates the relationships between wild and domestic species within the genus citrus. MBE. 2015; 10.1093/molbev/msv082 25873589 PMC4833069

[45] Shahid Masood M, Nishikawa T, Fukuoka S, Njenga PK, Tsudzuki T, Kadowaki K. The complete nucleotide sequence of wild rice (Oryza nivara) chloroplast genome: First genome wide comparative sequence analysis of wild and cultivated rice. Gene 2004, 340, 133–139. 10.1016/j.gene.2004.06.008 15556301

[46] Li DM, Zhao CY, Liu XF. Complete chloroplast genome sequences of *Kaempferia galanga* and *Kaempferia elegans*: molecular structures and comparative analysis. Molecules, 2019, 24,474. 10.3390/molecules24030474 PMC6385120 30699955

[47] Xu J, Chu Y, Liao B, Xiao S, Yin Q, Bai R, et al Panax ginseng genome examination for ginsenoside biosynthesis. Gigascience 2017; 6, 1–15. 10.1093/gigascience/gix093 PMC5710592 29048480

[48] Chen S, Xu J, Liu C, Zhu Y, Nelson DR, Zhou S, et al Genome sequence of the model medicinal mushroom Ganoderma lucidum. Nat. Commun. 2012; 3, 913 10.1038/ncomms1923 22735441 PMC3621433

[49] Raubeson LA, Peery R, Chumley TW, Dziubek C, Fourcade HM, Boorem JL, et al Comparative chloroplast genomics: Analyses including new sequences from the angiosperms Nuphar advena and Ranunculus macranthus. BMC Genom. 2007; 8, 174–201. 10.1186/1471-2164-8-174 PMC1925096 17573971

[50] Wang RJ, Cheng CL, Chang CC, Wu CL, Su TM, Chaw SM. Dynamics and evolution of the inverted repeat-large single copy junctions in the chloroplast genomes of monocots. BMC Evol. Biol. 2008; 8, 36–50. 10.1186/1471-2148-8-36 18237435 PMC2275221

