# Supplementary figures and images for: Complete chloroplast genome sequence and phylogenetic analysis of Spathiphyllum 'Parrish'
Source: PLoS One. 2019 Oct 23;14(10):e0224038. doi: 10.1371/journal.pone.0224038 (PMC6808432; doi:10.1371/journal.pone.0224038)

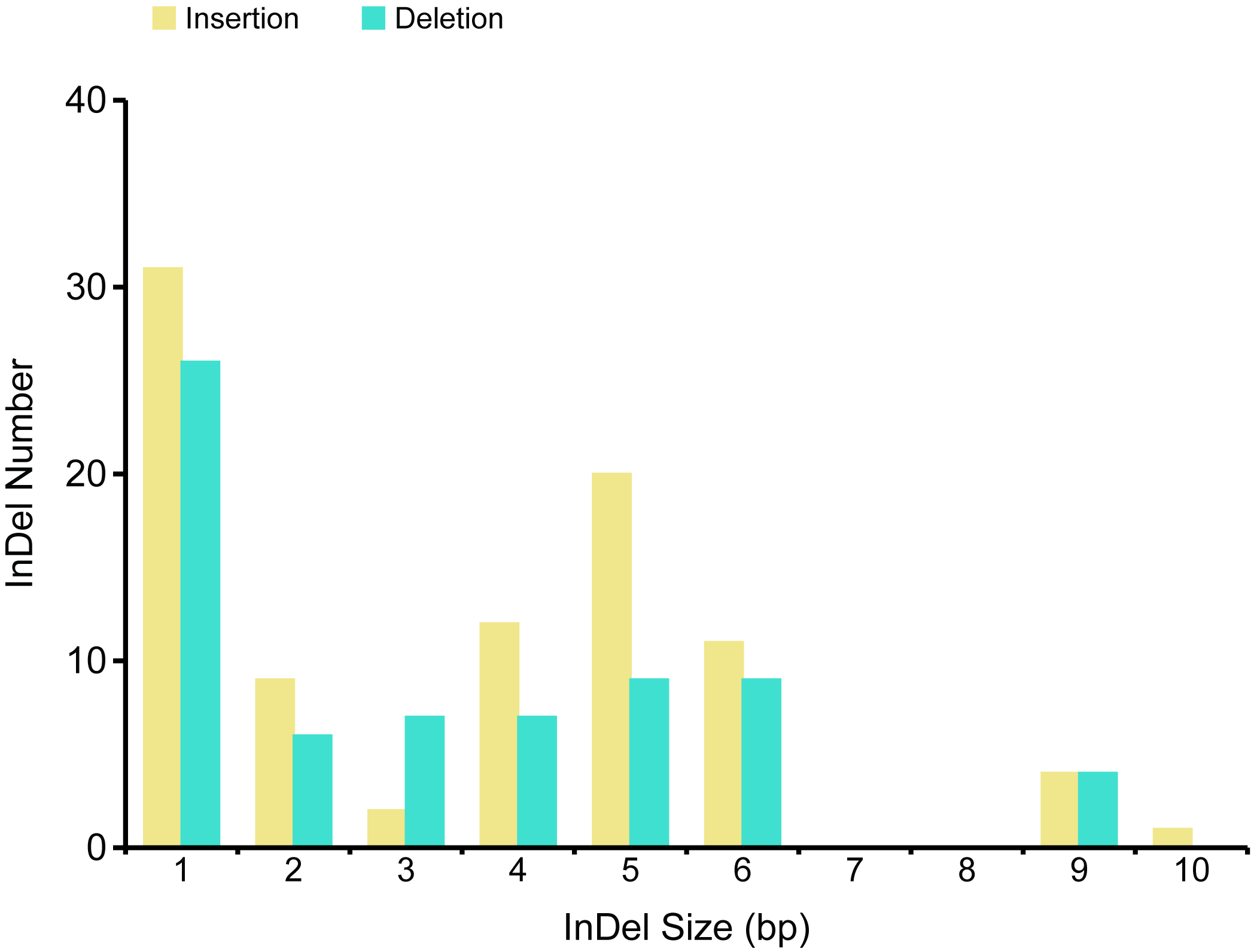

Supplement: S1 Fig — (TIF) [file pone.0224038.s001.tif]
